# Associations Between Annual Medicare Part D Low-Income Subsidy Loss and Prescription Drug Spending and Use

**DOI:** 10.1001/jamahealthforum.2023.5152

**Published:** 2024-02-02

**Authors:** Vicki Fung, Mary Price, David Cheng, Tej A. Patel, Zhiyou Yang, John Hsu, Margarita Alegria, Joseph P. Newhouse

**Affiliations:** 1 Massachusetts General Hospital, Boston; 2 Harvard Medical School, Boston, Massachusetts; 3 University of Pennsylvania, Philadelphia; 4 Harvard T.H. Chan School of Public Health, Boston, Massachusetts; 5 Harvard Kennedy School, Cambridge, Massachusetts; 6 National Bureau of Economic Research, Cambridge, Massachusetts

## Abstract

**Importance:**

The Medicare Part D Low Income Subsidy (LIS) program provides millions of beneficiaries with drug plan premium and cost-sharing assistance. The extent to which LIS recipients experience subsidy losses with annual redetermination cycles and the resulting associations with prescription drug affordability and use are unknown.

**Objective:**

To examine how frequently annual LIS benefits are lost among Medicare Part D beneficiaries and how this is associated with prescription drug use and out-of-pocket costs.

**Design, Setting, and Participants:**

In this cohort study of Medicare Part D beneficiaries from 2007 to 2018, annual changes in LIS recipients among those automatically deemed eligible (eg, due to dual eligibility for Medicare and Medicaid) and nondeemed beneficiaries who must apply for LIS benefits were analyzed using Medicare enrollment and Part D event data. Subsidy losses were classified in 4 groups: temporary losses (<1 year); extended losses (≥1 year); subsidy reductions (change to partial LIS); and disenrollment from Medicare Part D after subsidy loss. Temporary losses could more likely represent subsidy losses among eligible beneficiaries. Multinomial logit models were used to examine associations between beneficiary characteristics and subsidy loss; linear regression models were used to compare changes in prescription drug cost and use in the months after subsidy losses vs before. Analyses were conducted between November 2022 and November 2023.

**Exposure:**

Subsidy loss at the beginning of each year among subsidy recipients in December of the prior year.

**Main Outcomes and Measures:**

The main outcomes were out-of-pocket costs and prescription drug fills overall and for 4 classes: antidiabetes, antilipid, antidepressant, and antipsychotic drugs.

**Results:**

In 2008, 731 070 full LIS beneficiaries (17%) were not deemed automatically eligible (39% were aged <65 years; 59% were female). Nearly all beneficiaries deemed automatically eligible (≥99%) retained the subsidy annually from 2007 to 2018, compared with 78% to 84% of nondeemed beneficiaries. Among nondeemed beneficiaries, disabled individuals younger than 65 years and racial and ethnic minority groups were more likely to have temporary subsidy losses vs none. Temporary losses were associated with an average 700% increase in out-of-pocket drug costs (+$52.72/mo [95% CI, 52.52-52.92]) and 15% reductions in prescription fills (−0.58 fills/mo [95% CI, −0.59 to −0.57]) overall. Similar changes were found for antidiabetes, antilipid, antidepressant, and antipsychotic prescription drug classes. Beneficiaries who retained their subsidy had few changes.

**Conclusions and Relevance:**

The conclusions of this cohort study suggest that efforts to help eligible beneficiaries retain Medicare Part D subsidies could improve drug affordability, treatment adherence, and reduce disparities in medication access.

## Key Points

**Question:**

How frequently do beneficiaries lose Medicare Part D low-income subsidies (LIS), and is this associated with drug affordability and use?

**Findings:**

In this cohort study from 2007 to 2018, approximately one-fifth of those not deemed automatically eligible for LIS lost subsidies annually. Being younger than 65 years and a member of a racial and ethnic minority group was associated with increased temporary subsidy losses, out-of-pocket costs, and reduced prescription drug fills for chronic illnesses and overall.

**Meaning:**

Reducing barriers to maintaining Medicare Part D LIS could improve drug affordability and adherence, especially for disabled and racially and ethnically minoritized beneficiaries.

## Teaser

This cohort study examines how frequently annual low-income subsidy benefits are lost among Medicare Part D beneficiaries and how this is associated with prescription drug use and out-of-pocket costs.

## Introduction

The Medicare Part D prescription drug program provides premium and cost-sharing assistance for beneficiaries who qualify for the Low-Income Subsidy (LIS) program. In 2023, 13.4 million beneficiaries (27% of Medicare Part D enrollees) received full or partial LIS benefits.^1^ For beneficiaries receiving the full subsidy, the LIS provides complete assistance with Part D plan premiums and annual deductibles and reduces beneficiary cost-sharing after the deductible. Receiving this assistance is associated with reductions in cost-related treatment nonadherence and financial stress among beneficiaries with low incomes.^2,3,4,5^

The LIS is administered by the Centers for Medicare & Medicaid Services (CMS) and the Social Security Administration (SSA), which use an annual cycle to redetermine beneficiaries’ eligibility for the program. The extent to which beneficiaries experience subsidy losses with the redetermination process and the potential relationships with drug use remain unknown. Among beneficiaries who are dually eligible for Medicare and Medicaid, churn from Medicaid (temporary losses of coverage) is a frequent occurrence, more common among Black beneficiaries and those younger than 65 years who qualify for Medicare due to disability.^6,7^ Administrative barriers to maintaining Medicaid coverage were identified as a major contributor to coverage losses, particularly short-term temporary losses.^6^ Churn in Medicaid coverage has been associated with delaying necessary care, difficulty accessing care, and increases in potentially preventable hospitalizations.^8,9,10^

Loss of Medicare Part D LIS benefits could lead to substantial increases in out-of-pocket drug costs, which have been associated with reductions in adherence to medications for chronic illnesses, including for cardiometabolic diseases and mental health conditions, as well as adverse health outcomes, including worse disease control (eg, elevated hemoglobin A_1c_ levels) and hospitalizations.^11,12,13,14,15,16^ The annual redetermination process for the LIS benefit differs for beneficiaries who are automatically deemed eligible and beneficiaries who are not automatically deemed eligible and must apply for benefits (hereafter, nondeemed beneficiaries). Deemed beneficiaries include full and partial benefit dually eligible enrollees and those without Medicaid who receive Supplemental Security Income. Individuals who meet these criteria (based on state files submitted to the SSA between July and December) have their LIS benefits extended for the following calendar year. Nondeemed beneficiaries are eligible for the full subsidy if their incomes are below 135% of the federal poverty level, and their assets are below the program limit ($9090 for an individual in 2023). Beneficiaries with incomes below 150% of the federal poverty level and assets below $15 160 (in 2023) are eligible for partial assistance. In September, nondeemed LIS beneficiaries selected by SSA for recertification are contacted and have 30 days to return their paperwork.^17^

In this cohort study, we use Medicare data from 2007 to 2018 to characterize how frequently beneficiaries experience subsidy losses in the Part D LIS program; assess differences in the likelihood of subsidy loss by age, disability status, and race and ethnicity; and examine the association of subsidy loss with changes in prescription drug out-of-pocket costs and use, including for 4 chronic drug classes.

## Methods

### Study Population and Data Sources

This cohort study uses Medicare enrollment and Part D event data from 2007 to 2018 for a 50% sample of beneficiaries who received Part D LIS benefits during the study period and had continuous Medicare A and B enrollment for 12 months in a given year. Enrollment data include monthly enrollment in Medicare Parts A, B, D, and Medicare Advantage, dual eligibility for Medicare and Medicaid, and Part D LIS status. Part D event data include prescription drug fills covered by stand-alone Prescription Drug Plans and Medicare Advantage Prescription Drug Plans. The Mass General Brigham institutional review board determined that this project meets criteria for exemption 4, secondary research for which consent is not required. Analyses were conducted between November 2022 and November 2023.

The standard (unsubsidized) Part D benefit in 2018 included a $405 deductible and 25% coinsurance for brand and generic drugs during the initial coverage period. Recipients of the full subsidy receive coverage for benchmark Part D premiums; recipients also have no deductible and low copays during the initial coverage period (eg, maximum of $3.35 for generic and $8.35 for brand drugs in 2018). Individuals receiving the partial subsidy pay premiums on a sliding scale, have reduced deductibles, and 15% coinsurance during the initial coverage period. LIS recipients did not have the standard Part D coverage gap and have lower cost sharing during the catastrophic phase (eTable 1 in Supplement 1).^18^

### Subsidy Enrollment and Losses

We examined the number of beneficiaries receiving the full LIS each month from January 2008 through December 2018. To assess changes in subsidy status likely associated with the annual redetermination process, we identified individuals who received the full LIS in December of each year (2007-2017) and changes in subsidy that occurred at the beginning of the following year stratified by deemed LIS status. Among deemed beneficiaries, we also separately examined data from individuals with full and partial benefit dual eligibility (eTable 2 in Supplement 1). Part D plan sponsors can offer a 3-month grace period for individuals who no longer qualify for LIS but have applied for benefits^17^; therefore, we focus on subsidy changes between January through April of each year.

Among nondeemed beneficiaries for whom redetermination was not automatic, we classified subsidy losses into 4 types: (1) temporary losses (ie, loss of the full LIS followed by regaining the subsidy for at least 1 month during the calendar year); (2) extended losses (ie, loss of the full LIS without regaining the subsidy during the calendar year); (3) subsidy reductions (ie, change from full to partial LIS); and (4) disenrollment from Medicare Part D after subsidy loss (ie, loss of full LIS followed by dropping Part D coverage during the calendar year).

Beneficiaries with a subsidy reduction were determined by SSA to meet eligibility criteria for a partial subsidy, rather than the full subsidy (perhaps due to increases in income or assets). For the other groups, we could not identify beneficiaries who lost their subsidy due to eligibility changes vs those who remained eligible but did not retain their subsidy.

We examined if age, disability status, and race and ethnicity were associated with the 4 different types of subsidy loss or retaining the subsidy from year to year. Age was classified as younger than 65 years, 65 to 79 years, and 80 years and older. Nearly all beneficiaries younger than 65 years qualify for Medicare due to disability, which was determined based on receipt of Social Security Disability Insurance. The Medicare Return to Intermediary variable for race and ethnicity includes 7 categories: American Indian or Alaska Native, Asian, Black (or African American), Hispanic, Non-Hispanic White, other, and unknown.^19,20^

### Statistical Analysis

We fit a multinomial logistic regression model for yearly subsidy status (retained subsidy, temporary loss, extended loss, reduced subsidy, or disenrollment from Medicare Part D) using generalized estimating equations to assess associations with beneficiary characteristics, while accounting for correlated outcomes within beneficiaries. An independent structure was used for the working correlation matrix. These models additionally adjusted for quintile of prior year drug costs to account for potential need for Part D coverage, whether the beneficiary lived in a different state in the prior year, as moving could necessitate changing Part D plans, whether the beneficiary was enrolled in Traditional Medicare vs Medicare Advantage in January of each year, and state of residence. We report adjusted marginal probabilities of each type of subsidy loss for each age and racial and ethnic group.

### Prescription Drug Use Outcomes

We examined the association between subsidy losses and out-of-pocket drug costs and the number of prescription drug fills standardized to a 30-day supply. We examined these outcomes for all prescription drugs and for 4 chronic drug classes that are commonly used among Medicare beneficiaries: antidiabetes, antilipid, antidepressant, and antipsychotic medications (eTable 3 in Supplement 1).^21^ In secondary analyses, we examined insulin and noninsulin diabetes drugs separately. These analyses were limited to those who received LIS benefits for 12 months in the year before losing their subsidy (81% of the overall cohort in 2008). For analyses examining changes in the use of specific drug classes, beneficiaries were required to have at least 1 fill of a drug in the following classes in the prior year: antidiabetes (23%), antilipid (40%), antidepressant (28%), and antipsychotic (9%) medications in 2008 (see eTable 4 in Supplement 1).

To compare changes in monthly drug use and costs before subsidy losses vs after, we used linear regression models stratified by type of subsidy loss that included an indicator for months with the full subsidy vs without, adjusted for calendar year, enrollment in traditional Medicare vs Medicare Advantage, and fixed effects at the beneficiary level to assess within-person changes in outcomes. Models included a separate indicator for months after regaining the subsidy for those with temporary losses. For those who retained their subsidy, models compared adjacent calendar years. Beneficiaries who disenrolled from Part D were excluded from these analyses because we cannot observe prescription drug use during months when individuals were not enrolled in Medicare Part D. An expected percentage change in costs and fills in months before subsidy loss vs after was calculated by the ratio of the estimated change after subsidy loss to the unadjusted means prior to the loss.

## Results

### Medicare Part D LIS Enrollment Patterns

**Figure 1.**
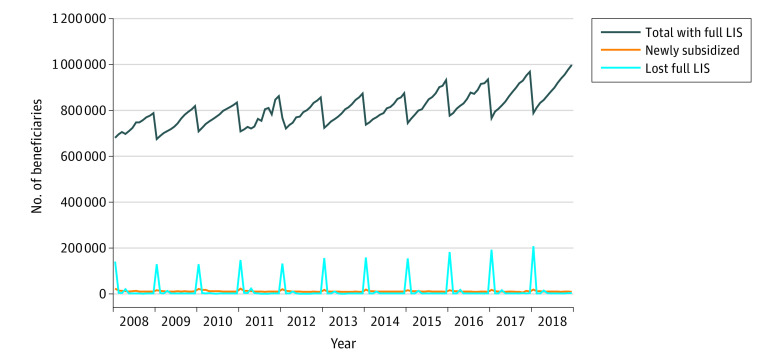
Total, New, and Loss of Enrollment in Nondeemed Full Low-Income Subsidy Recipients by Month Total with full low-income subsidy (LIS) reflects total number of beneficiaries receiving the full LIS in each month. Newly subsidized includes those receiving the full LIS who did have the full LIS in the prior month. Lost full LIS includes those who were receiving the full LIS in the prior month who did not have the full LIS in the current month.

Among 4 217 523 beneficiaries with the full LIS benefit in December 2007, 3 486 453 (83%) were deemed eligible and 731 070 (17%) were nondeemed (Table 1). The number of full LIS beneficiaries grew to 5 734 209 in December 2017, including 862 319 nondeemed beneficiaries (15%). For deemed beneficiaries, the annual percentage who retained the full subsidy across years ranged from 99% to 100% between 2007 and 2018; for nondeemed beneficiaries, this percentage reached a high of 84% in 2012 and a low of 78% in 2018 (Table 1). Total monthly enrollment of nondeemed full LIS recipients demonstrates a sawtooth pattern, with steady increases in enrollment throughout each calendar year followed by decreasing enrollment in January, consistent with the annual redetermination cycle (Figure 1).

**Table 1.** Percentage Full Low-Income Subsidy Recipients in December of Prior Year

Year	Nondeemed beneficiaries		Deemed beneficiaries^a^	
	Full LIS benefit in December of prior year, No.	Retained full LIS from January to April of current year, %	Full LIS benefit in December of prior year, No.	Retained full LIS from January to April of current year, %
2008	731 070	81	3 486 453	99
2009	699 006	82	3 636 825	99
2010	729 008	84	3 745 560	99
2011	740 480	79	3 939 508	99
2012	767 603	84	4 096 473	99
2013	762 749	81	4 270 816	99
2014	781 165	81	4 392 294	99
2015	783 313	81	4 553 912	99
2016	837 158	79	4 658 507	100
2017	835 030	78	4 777 768	100
2018	862 319	78	4 871 890	100

Abbreviation: LIS, low-income subsidy.

^a^ Deemed beneficiaries include full and partial benefit dual-eligible beneficiaries and those without Medicaid who receive Supplemental Security Income.

### Characteristics of Nondeemed Full LIS Recipients

Among nondeemed full LIS recipients in December 2007, 39% were younger than 65 years; 59% were female; 24% were enrolled in Medicare Advantage; and 1% were American Indian/Alaska Native, 2% were Asian, 21% were Black, 12% were Hispanic, and 63% were White (Table 2). In 2017, the proportion who were members of racial and ethnic minority groups increased (eg, 4% Asian and 16% Hispanic), as did the proportion enrolled in Medicare Advantage (45%).

**Table 2.** Characteristics of Nondeemed Enrollees With Medicare Part D Low-Income Subsidy (LIS) Benefit

Variable	%	
	2008	2018
Total with subsidy in December of prior year, No.^a^	731 070	862 319
Age, y		
<65	39	38
65-79	43	48
≥80	18	14
Original reason for entitlement		
Age	50	46
Disability	49	53
Sex		
Female	59	57
Male	41	43
Race and ethnicity		
American Indian or Alaska Native	1	1
Asian	2	4
Black	21	20
Hispanic	12	16
White	63	58
Other or unknown^b^	1	2
Lived in a different state in prior year	2	2
Enrolled in MA vs TM^c^	24	45
LIS status in current year		
Retained full LIS for 12 mo	81	78
Temporary loss of LIS	5	5
Extended loss of LIS	10	11
Reduced subsidy (partial LIS)	2	2
Disenrolled from Part D after loss of LIS	2	5

Abbreviations: LIS, low-income subsidy; MA, Medicare Advantage; TM, traditional Medicare.

^a^ Characteristics among nondeemed beneficiaries with full LIS in December 2007 and December 2018. A total of 10 951 and 11 678 beneficiaries had an original reason for entitlement of end-stage kidney disease in 2008 and 2018.

^b^ Other was a distinct category provided by Medicare data.

^c^ In January of current year.

Among nondeemed full LIS recipients in December 2007, 10% had extended subsidy losses in 2008 (Table 2), 5% had temporary losses, 2% had subsidy reductions, and 2% had disenrolled from Medicare Part D after losing their subsidy (individuals who were disenrolling grew to 5% in 2018; eTable 5 in Supplement 1). Beneficiaries with temporary losses had a mean (SD) of 3.3 (3.0) months without subsidy (eTable 6 in Supplement 1).

### Subsidy Losses by Age and Race and Ethnicity

**Figure 2.**
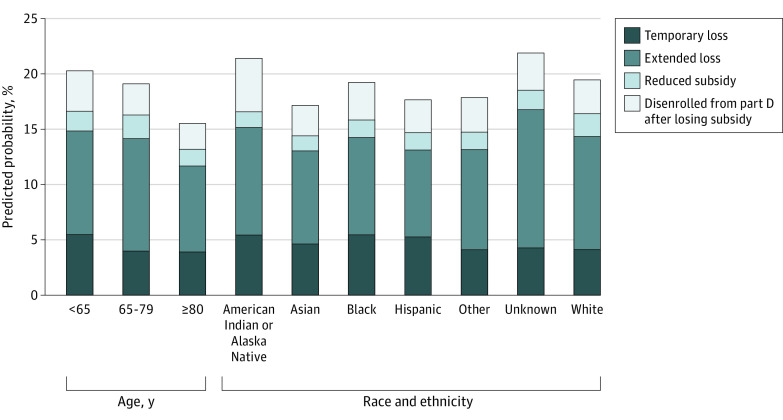
Adjusted Marginal Probability of Annual Change in Subsidy Status by Age Group and Race and Ethnicity in Nondeemed Full Low-Income Subsidy (LIS) Recipients This figure displays the marginal probability of having an annual change in subsidy loss (vs retaining subsidy) for each group based on a multinomial logistic regression comparing each subsidy group to the group that does not lose the full LIS. The model is also adjusted for sex, quintile of prior year drug costs, an indicator for moving states, enrollment in Medicare Advantage vs traditional Medicare in January, year, and state. Other race and ethnicity is a distinct category reported in Medicare data.

In multivariable analyses, recipients of LIS younger than 65 years vs 65 to 79 years of age were more likely to experience temporary subsidy losses (marginal probability of 5.5% vs 4.0%; odds ratio [OR], 1.40 [95% CI, 1.39-1.41]) and to disenroll from Part D after subsidy loss (3.7% vs 2.8%; OR, 1.35 [95% CI, 1.34-1.36]), rather than retain their subsidy (79.6% vs 80.9%, Figure 2; eTable 7 in Supplement 1). In contrast, beneficiaries younger than 65 years vs 65 to 79 years of age were less likely to have subsidy reductions or extended subsidy losses. Beneficiaries 80 years and older vs 65 to 79 years of age were less likely to experience any subsidy loss.

American Indian or Alaska Native, Asian, Black, and Hispanic beneficiaries were more likely to experience temporary subsidy losses vs retain their subsidy, compared with White beneficiaries (5.5% vs 4.1%; OR, 1.32 [95% CI, 1.31-1.33] for Black beneficiaries compared with White beneficiaries). Beneficiaries of American Indian or Alaska Native and Black race and ethnicity were more likely to disenroll from Medicare Part D after subsidy loss compared with White beneficiaries; however, Asian and Hispanic beneficiaries were less likely to experience such changes. In addition, beneficiaries of racial and ethnic minority groups were less likely to have subsidy reductions or extended subsidy losses compared with White beneficiaries.

### Changes in Out-of-Pocket Costs and Prescription Drug Fills

Beneficiaries who retained their subsidy had little to no change in monthly out-of-pocket costs or prescription drug fills across years. Beneficiaries who retained their subsidy had mean out-of-pocket costs of $9.14 and filled an average of 4.2 prescription drug fills per month in the previous year. Annual changes ranged from a 3% decrease in out-of-pocket spending for antipsychotics to a 2% increase for all prescription drugs, with similar changes observed in prescription drug fills (Table 3).

**Table 3.** Change in Monthly Out-of-Pocket Spending and Prescription Drug Fills by Low-Income Subsidy (LIS) Benefit Status

LIS status	Out-of-pocket costs, $				
	Drug class	Mean from prior period	Months after vs before losing subsidy		*P* value
			Change, %	Adjusted change (95% CI)	
No loss^a^	All	9.14	2	0.17 (0.16 to 0.18)	<.001
	Antidiabetes	2.96	0	0.01 (0.00 to 0.01)	.05
	Antilipids	1.77	0	0.00 (0.00 to 0.01)	.002
	Antidepressants	1.63	−3	−0.04 (−0.05 to −0.04)	<.001
	Antipsychotics	2.27	−3	−0.07 (−0.09 to −0.06)	<.001
Temporary loss^b^	All	7.53	700	52.72 (52.52 to 52.92)	<.001
	Antidiabetes	2.56	746	19.11 (18.96 to 19.26)	<.001
	Antilipids	1.36	411	5.58 (5.54 to 5.62)	<.001
	Antidepressants	1.38	484	6.69 (6.63 to 6.74)	<.001
	Antipsychotics	2.00	1770	35.47 (35.03 to 35.91)	<.001
Extended loss	All	6.99	578	40.42 (40.26 to 40.58)	<.001
	Antidiabetes	2.40	675	16.18 (16.03 to 16.33)	<.001
	Antilipids	1.36	403	5.46 (5.42 to 5.50)	<.001
	Antidepressants	1.37	460	6.31 (6.26 to 6.36)	<.001
	Antipsychotics	1.91	1179	22.48 (22.06 to 22.90)	<.001
Reduced subsidy (full to partial LIS)^c^	All	9.92	196	19.48 (19.31 to 19.65)	<.001
	Antidiabetes	2.86	351	10.04 (9.88 to 10.20)	<.001
	Antilipids	1.71	156	2.67 (2.63 to 2.72)	<.001
	Antidepressants	1.57	148	2.32 (2.27 to 2.38)	<.001
	Antipsychotics	2.18	487	10.64 (10.24 to 11.04)	<.001
**LIS status**	**30-d Prescription drug fills, No.**				
No loss^a^	All	4.2	2	0.096 (0.094 to 0.099)	<.001
	Antidiabetes	1.3	0	0.003 (0.001 to 0.005)	.001
	Antilipids	0.8	−1	−0.013 (−0.014 to −0.011)	<.001
	Antidepressants	0.9	−3	−0.029 (−0.030 to −0.028)	<.001
	Antipsychotics	0.9	−4	−0.036 (−0.038 to −0.034)	<.001
Temporary loss^b^	All	4.0	−15	−0.580 (−0.59 to −0.571)	<.001
	Antidiabetes	1.2	−21	−0.255 (−0.263 to −0.248)	<.001
	Antilipids	0.8	−16	−0.126 (−0.131 to −0.122)	<.001
	Antidepressants	0.9	−14	−0.120 (−0.124 to −0.115)	<.001
	Antipsychotics	0.8	−19	−0.150 (−0.156 to −0.143)	<.001
Extended loss	All	3.9	−15	−0.603 (−0.608 to −0.598)	<.001
	Antidiabetes	1.2	−21	−0.255 (−0.26 to −0.251)	<.001
	Antilipids	0.8	−17	−0.143 (−0.145 to −0.14)	<.001
	Antidepressants	0.9	−18	−0.161 (−0.164 to −0.158)	<.0001
	Antipsychotics	0.8	−29	−0.239 (−0.244 to −0.235)	<.0001
Reduced subsidy (full to partial LIS)^c^	All	5.1	−9	−0.446 (−0.46 to −0.433)	<.001
	Antidiabetes	1.3	−14	−0.182 (−0.191 to −0.172)	<.001
	Antilipids	0.9	−10	−0.089 (−0.094 to −0.083)	<.001
	Antidepressants	0.9	−10	−0.091 (−0.098 to −0.085)	<.001
	Antipsychotics	0.9	−23	−0.207 (−0.217 to −0.196)	<.001

^a^ For those with no subsidy loss, models compared adjacent calendar years; if beneficiaries retained their subsidy over multiple years, we used their first observed full year with the subsidy as the preperiod and classified subsequent years such that the same calendar year were not both a preperiod and postperiod.

^b^ Includes months with partial LIS. The coefficient for months in the postperiod with full LIS coverage regained vs months in the preperiod with full LIS coverage can be found in eTable 9 in Supplement 1.

^c^ The coefficient for months in the postperiod with no LIS vs months in the preperiod with full LIS coverage can be found in eTable 9 in Supplement 1.

In contrast, beneficiaries with temporary subsidy losses had an average monthly out-of-pocket increase of 700% (plus $52.72 [95% CI, 52.52-52.92]) and 15% reduction in prescription drug fills in the months without subsidy vs with (−0.58 fills [95% CI, −0.59 to −0.57]). Among beneficiaries with temporary losses, monthly spending increases ranged from $5.58 (95% CI, 5.54-5.62) for antilipid drugs to $35.47 (95% CI, 35.03-35.91) for antipsychotic drugs; reductions in drug use ranged from 0.26 fills/mo (95% CI, −0.26 to −0.25) for antidiabetes drugs to 0.12 fills/mo (95% CI, −0.124 to −0.115) for antidepressants. Beneficiaries with extended subsidy losses and subsidy reductions also had reductions in out-of-pocket costs and prescription drugs fills in the months without full subsidy vs with (Table 3; eTable 8 in Supplement 1).

## Discussion

In this cohort study, we found that the number of beneficiaries enrolled in Medicare Part D with full LIS increased substantially over time. Nearly all deemed beneficiaries, who automatically requalify for their LIS in the next year based on state reports, maintained their subsidy across years. In contrast, approximately 1 in 5 nondeemed full subsidy recipients, who had to recertify their LIS eligibility on their own, lost it for at least part of the year, faced large increases in out-of-pocket prescription drug costs after subsidy loss, and reduced their medication use overall and for chronic illnesses during months without the subsidy benefit.

The limited evidence on Medicare Part D LIS enrollment patterns suggests that subsidy take-up rates are poor among nondeemed beneficiaries (only 30% of eligible beneficiaries enrolled).^22^ Annual subsidy losses could contribute to poor take-up if those who lose their subsidies do not reapply for benefits. In this study, approximately half of those losing their subsidy had extended losses that lasted longer than a calendar year. Importantly, the proportion of these beneficiaries who remained eligible for the subsidy (vs those who were no longer eligible due to positive changes in their income or assets) was unable to be determined in our study. Approximately one-quarter of those with subsidy losses had more temporary gaps in their LIS enrollment. The short duration of these subsidy losses (3 months, on average) raises concerns about these beneficiaries being continuously eligible for benefits yet facing barriers to remaining enrolled.

Beneficiaries with both temporary and extended losses faced large increases in out-of-pocket costs and reduced prescription drug use in months without LIS vs with LIS. Similar decreases were found in the use of antidiabetes, antilipid, antidepressant, and antipsychotic prescription drugs. These findings are consistent with a substantial body of evidence linking higher out-of-pocket costs with decreased use of clinically necessary medications^23,24,25,26^ and raise concerns about the adverse clinical consequences of subsidy losses with reductions in medication adherence.^11,12^ Moreover, the evidence suggests that ineligibility for cost-sharing subsidies is associated with greater cost-related barriers to care among racial and ethnic minority beneficiaries.^27^ Thus, examining variation in the consequences of LIS subsidy losses on drug use by race and ethnicity and disability status is critical for understanding the potential implications of such disruptions on health equity.

A smaller but growing percentage of full LIS recipients (2%-5%) disenrolled from Part D coverage altogether after losing their subsidy. The loss of Part D coverage is especially concerning if these beneficiaries had no other source of prescription drug coverage, such as through the Department of Veterans Affairs.^28^ Approximately half of beneficiaries without Medicare Part D have no other source of comparable prescription drug coverage. Moreover, beneficiaries could also face financial penalties to reenroll in Part D, unless the beneficiaries qualify for LIS at re-enrollment.

Approximately 2% of full subsidy recipients had a subsidy reduction from full to partial assistance, which was associated with increases in out-of-pocket costs and reductions in prescription drug use. At the maximum income limit, individuals qualifying for partial subsidies had annual incomes of $20 385 compared with $18 347 for the full subsidy in 2022. Overall, only approximately 3% of Medicare Part D LIS recipients received partial vs full assistance in 2019.^29^ The Inflation Reduction Act of 2022 expands full assistance to those currently eligible for partial assistance starting in 2024, which will smooth these subsidy fluctuations and could help beneficiaries maintain adherence to prescription drug treatment.^30^

Administrative burdens associated with enrolling and maintaining enrollment in public programs have been found to contribute to gaps in assistance among eligible populations and disparities.^31,32,33^ We found that temporary gaps in Medicare Part D LIS assistance were more common among beneficiaries with disabilities younger than 65 years, as well as racial and ethnic minority groups. These findings are consistent with evidence that structural barriers, such as poor access to transportation, support, and language-concordant information, add to the administrative complexity for these groups when enrolling in public assistance programs.^34,35,36^ Losses of Medicaid coverage associated with the unwinding of the COVID-19 Public Health Emergency continuous Medicaid coverage requirement by states could have a cascading effect on LIS enrollment and prescription drug use because Medicare beneficiaries losing Medicaid benefits would no longer be automatically enrolled in the Medicare Part D LIS. Recent unwinding data reported by CMS suggest that more than one-third of Medicaid enrollees who completed a renewal were disenrolled from Medicaid, largely owing to procedural reasons.^37^ In August 2023, CMS issued requirements to states to reduce potential erroneous procedural denials.^38^

CMS also finalized federal rules to streamline Medicare Savings Program enrollment in November 2023. These rules include provisions to improve information exchange and coordination between the SSA and state Medicaid programs to coordinate applications and eligibility criteria for the Part D LIS and Medicare Savings Program, which could help low-income beneficiaries enroll and reenroll in the LIS through automatic deeming.^39,40^ Improving data exchange across programs serving low-income Medicare beneficiaries could increase historically low take-up rates of available subsidies^41^ and renewal rates, as demonstrated with automated Medicaid renewals.^42,43^ Identifying and addressing barriers to LIS renewal will be critical for improving the continuity of financial assistance and affordability of prescription medications, particularly for beneficiaries with a disability or who are members of racial and ethnic minority groups.

### Limitations

This was an observational study that examined associations, not causation; additionally, we acknowledge that some factors were unable to be observed. We were unable to identify which beneficiaries were selected for redetermination or why they lost their LIS status, such as actual changes in eligibility vs process-based rejections or nonresponse. For beneficiaries who disenrolled from Medicare Part D after losing their LIS, we could not examine changes in prescription drug use with Medicare claims data. Lastly, we did not have access to Medicare Advantage encounter data to examine the outcomes of subsidy losses on adverse clinical events, such as potentially preventable hospitalizations.

## Conclusions

Between 2008 and 2018, approximately 1 in 5 nondeemed beneficiaries lost their Medicare Part D LIS, while nearly all deemed beneficiaries retained their subsidy annually. Among nondeemed beneficiaries, temporary subsidy losses were common, suggestive of coverage losses among eligible beneficiaries, particularly disabled beneficiaries younger than 65 years and members of racial and ethnic minority groups. Moreover, subsidy losses were associated with reductions in overall prescription drug use and for 4 chronic drug classes, raising concerns about the adverse clinical outcomes of discontinuities in subsidy receipt. Efforts to help beneficiaries retain Medicare Part D subsidies are critical for improving drug affordability and adherence among low-income beneficiaries and reducing disparities in medication access.

## Notes

Supplement 1. **eTable 1.** Standard Part D Benefit Design for Those Receiving and Not Receiving the LIS in 2008 and 2018 **eTable 2.** Percent of Beneficiaries with Full LIS in Each Year Among Those With Full LIS in December of Prior Year Among Partial vs Full Benefit Dual-Eligible Beneficiaries (Deemed Eligible for LIS) **eTable 3.** Drug Class Definitions for Diabetes, Lipid, Antidepressant, and Antipsychotic Drugs **eTable 4.** Sample Sizes for Drug Use Analyses in 2008 and 2018 **eTable 5.** Percent of Beneficiaries With Each Type of Subsidy Loss Among Nondeemed Full LIS **eTable 6.** Mean Months With Full, Partial, or No Subsidy or Part D by Subsidy Loss Categories **eTable 7.** Likelihood of having an Annual Change in Subsidy Status by Age Group and Race and Ethnicity Among Nondeemed Full LIS recipients (Data Correspond to Figure 2) **eTable 8.** Secondary Analyses: Change in Monthly Out-of-Pocket Spending and Drug Fills After vs Before Losing Full Part D LIS Among Nondeemed Beneficiaries for Insulin and Noninsulin Diabetes Drugs **eTable 9.** Change in Drug Use, Cost, and Out-of-Pocket Spending After vs Before Losing Full Part D LIS; Additional Results for the Reduced Subsidy and Temporary Loss Groups

Supplement 2.
Data Sharing Statement
