# Supplementary material for: Associations Between Annual Medicare Part D Low-Income Subsidy Loss and Prescription Drug Spending and Use
Source: JAMA Health Forum. 2024 Feb 2;5(2):e235152. doi: 10.1001/jamahealthforum.2023.5152 (PMC10837747; doi:10.1001/jamahealthforum.2023.5152)
Supplement: Supplement 1. — eTable 1. Standard Part D Benefit Design for Those Receiving and Not Receiving the LIS in 2008 and 2018 eTable 2. Percent of Beneficiaries with Full LIS in Each Year Among Those With Full LIS in December of Prior Year Among Partial vs Full Benefit Dual-Eligible Beneficiaries (Deemed Eligible for LIS) eTable 3. Drug Class Definitions for Diabetes, Lipid, Antidepressant, and Antipsychotic Drugs eTable 4. Sample Sizes for Drug Use Analyses in 2008 and 2018 eTable 5. Percent of Beneficiaries With Each Type of Subsidy Loss Among Nondeemed Full LIS eTable 6. Mean Months With Full, Partial, or No Subsidy or Part D by Subsidy Loss Categories eTable 7. Likelihood of having an Annual Change in Subsidy Status by Age Group and Race and Ethnicity Among Nondeemed Full LIS recipients (Data Correspond to Figure 2) eTable 8. Secondary Analyses: Change in Monthly Out-of-Pocket Spending and Drug Fills After vs Before Losing Full Part D LIS Among Nondeemed Beneficiaries for Insulin and Noninsulin Diabetes Drugs eTable 9. Change in Drug Use, Cost, and Out-of-Pocket Spending After vs Before Losing Full Part D LIS; Additional Results for the Reduced Subsidy and Temporary Loss Groups [file jamahealthforum-e235152-s001.pdf]

Supplemental Online Content

Fung V, Price M, Cheng D, et al. Associations between annual Medicare Part D low-income subsidy loss and prescription drug spending and use. *JAMA Health Forum*. 2023;5(2):e235152. doi:10.1001/jamahealthforum.2023.5152

eTable 1. Standard Part D Benefit Design for Those Receiving and Not Receiving the LIS in 2008 and 2018

eTable 2. Percent of Beneficiaries with Full LIS in Each Year Among Those With Full LIS in December of Prior Year Among Partial vs Full Benefit Dual-Eligible Beneficiaries (Deemed Eligible for LIS)

eTable 3. Drug Class Definitions for Diabetes, Lipid, Antidepressant, and Antipsychotic Drugs

eTable 4. Sample Sizes for Drug Use Analyses in 2008 and 2018

eTable 5. Percent of Beneficiaries With Each Type of Subsidy Loss Among Nondeemed Full LIS

eTable 6. Mean Months With Full, Partial, or No Subsidy or Part D by Subsidy Loss Categories

eTable 7. Likelihood of having an Annual Change in Subsidy Status by Age Group and Race and Ethnicity Among Nondeemed Full LIS recipients (Data Correspond to Figure 2)

eTable 8. Secondary Analyses: Change in Monthly Out-of-Pocket Spending and Drug Fills After vs Before Losing Full Part D LIS Among Nondeemed Beneficiaries for Insulin and Noninsulin Diabetes Drugs

eTable 9. Change in Drug Use, Cost, and Out-of-Pocket Spending After vs Before Losing Full Part D LIS; Additional Results for the Reduced Subsidy and Temporary Loss Groups

This supplemental material has been provided by the authors to give readers additional information about their work.

© 2024 Fung V et al. *JAMA Health Forum.*

eTable 1. Standard Part D Benefit Design for those receiving and not receiving the LIS in 2008 and 2018

2008	Standard Benefit	Full LIS	Partial LIS
Deductible	$275	$0	$56
Initial coverage phase	25% coinsurance	≤$2.25 generic copay<br>≤$5.60 brand copay	15% coinsurance
Coverage gap (after $2,510 in total drug spending)	100% coinsurance	≤$2.25 generic copay<br>≤$5.60 brand copay	15% coinsurance
Catastrophic coverage period (after $4,050 in out-of-pocket drug spending)	5% coinsurance	$0	$2.25 generic copay<br>$5.60 brand copay
**2018**	**Standard Benefit**	**Full LIS**	**Partial LIS**
Deductible	$405	$0	$83
Initial coverage phase	25% coinsurance	≤$3.35 generic copay<br>≤$8.35 brand copay	15% coinsurance
Coverage gap (after $3,750 in total drug spending)	44% generic coinsurance<br>25% brand coinsurance	≤$3.35 generic copay<br>≤$8.35 brand copay	15% coinsurance
Catastrophic coverage period (after $5,000 in out-of-pocket drug spending)	5% coinsurance	$0	$3.35 generic copay<br>$8.35 brand copay

© 2024 Fung V et al. *JAMA Health Forum.*

eTable 2. Percent of beneficiaries with Full LIS in each year among those with Full LIS in December of prior year among partial vs. full benefit dual-eligible beneficiaries (deemed eligible for LIS)

	Deemed beneficiaries			
	Partial Benefit Dual-eligible		Full Benefit Dual-eligible	
	No. with Full LIS in Dec of prior year	% that Retained Full LIS Jan-Apr of current year	No. with Full LIS in Dec of prior year	% that Retained Full LIS Jan-Apr of current year
2008	823,174	97%	2,663,279	100%
2009	889,781	97%	2,747,044	100%
2010	963,908	97%	2,781,652	100%
2011	1,079,657	98%	2,859,851	100%
2012	1,186,933	98%	2,909,540	100%
2013	1,255,962	98%	3,014,854	100%
2014	1,310,151	98%	3,082,143	100%
2015	1,350,414	98%	3,203,498	100%
2016	1,386,606	99%	3,271,901	100%
2017	1,451,434	99%	3,326,334	100%
2018	1,461,214	99%	3,410,676	100%

© 2024 Fung V et al. *JAMA Health Forum.*

eTable 3. Drug class definitions for diabetes, lipid, antidepressant, and antipsychotic drugs

Antidiabetes drugs (First databank)	Antilipid drugs (First databank)
Agents to treat Hypoglycemia (Hyperglycemics)	Antihyperlipidemic - ATP-Citrate Lyase (ACLY) Inhibitor
Antidiabetic - CD3 Directed Monoclonal Antibody	Antihyperlipidemic - Angiopoietin-like 3 (ANGPTL3) Inhibitor, MAb
Antihyperglycemic - Alpha-Glucosidase Inhibitors	Antihyperlipidemic - Anti-PCSK9 Monoclonal Antibody
Antihyperglycemic - DPP-4 Inhibitors & HMG CoA Reduct.Inhib (statins)	Antihyperlipidemic - Bile Acid Sequestrants
Antihyperglycemic - Dipeptidyl Peptidase-4 (DPP-4) Inhibitors	Antihyperlipidemic - Fibric Acid Derivatives
Antihyperglycemic - Dopamine Receptor Agonists	Antihyperlipidemic - HMG CoA Reductase Inhibitor & Niacin Combinations
Antihyperglycemic - Dual GIP and GLP-1 Receptor Agonists	Antihyperlipidemic - HMG CoA Reductase Inhibitors (statins)
Antihyperglycemic - Glucagon-Like Peptide-1 (GLP-1) Receptor Agonists	Antihyperlipidemic - Nicotinic Acid Derivatives
Antihyperglycemic - Glucocorticoid (Cortisol) Receptor Blocker (GR-II)	Antihyperlipidemic - Omega-3 Fatty Acid Type
Antihyperglycemic - Meglitinide Analog and Biguanide Combinations	Antihyperlipidemic - PCSK9 Inhibitors
Antihyperglycemic - Meglitinide Analogs	Antihyperlipidemic - Selective Cholesterol Absorption Inhibitor
Antihyperglycemic - SGLT-2 Inhibitor & Biguanide Combinations	Antihyperlipidemic - Thyroid Hormones-Analogs
Antihyperglycemic - SGLT-2 Inhibitor & DPP-4 Inhibitor Combinations	Antihyperlipidemic - apolipoprotein B-100 Synthesis Inhibitor
Antihyperglycemic - SGLT-2 Inhibitor and Biguanide Combinations	Antihyperlipidemic Agents - Dietary Source
Antihyperglycemic - SGLT-2 Inhibitor and DPP-4 Inhibitor Combinations	Antihyperlipidemic Agents - Dietary Source Combinations
Antihyperglycemic - Sodium Glucose Cotransporter-2 (SGLT2) Inhibitors	Antihyperlipidemic HMG CoA Reduct Inhib & Calcium Channel Blocker Comb
Antihyperglycemic - Sulfonylurea Derivatives	Antihyperlipidemic HMG CoA Reduct Inhib and Calcium Channel Blocker
Antihyperglycemic - Sulfonylurea and Biguanide Combinations	Antihyperlipidemic- ATP-Citrate Lyase and Cholesterol Absorption Inhib
Antihyperglycemic - Thiazolidinedione and Biguanide Combinations	Antihyperlipidemic-HMG CoA Reduct Inhib & Cholesterol Absorption Inhib
Antihyperglycemic - Thiazolidinedione and Sulfonylurea Combinations	Antihyperlipidemic-HMG CoA Reduct Inhib and Cholesterol Absorp Inhibit
Antihyperglycemic, Amylin Analog-Type	Antihyperlipidemic-HMG CoA Reductase Inhibitor-Aspirin, Buffered Comb.
Antihyperglycemic, Incretin Mimetic,GLP-1 Receptor Agonist Analog-Type	Antihyperlipidemic-Microsomal Triglyceride Transfer Protein (MTP)Inhib
Antihyperglycemic-Dipeptidyl Peptidase-4 (DPP-4) Inhibitor & Biguanide	

© 2024 Fung V et al. *JAMA Health Forum.*

Antihyperglycemic-Dipeptidyl Peptidase-4 Inhibitor & Thiazolidinedione	
Antihyperglycemic-Dipeptidyl Peptidase-4(DPP-4)Inhibitor and Biguanide	
Antihyperglycemic-Insulin, Long Acting and GLP-1 Receptor Agonist Comb	
Antihyperglycemic-SGLT-2 inhibitor, DPP-4 inhibitor and Biguanide comb	
Antiparkinson Therapy - Non-ergot Dopamine Agonist Agents	
Human Insulins - Fixed Combinations	
Human Insulins - Intermediate Acting	
Human Insulins - Long Acting	
Human Insulins - Rapid Acting	
Human Insulins - Short Acting	
Insulin - Beef	
Insulin - Beef and Pork Combinations	
Insulin - Pork	
Insulin Analogs - Fixed Combinations	
Insulin Analogs - Long Acting	
Insulin Analogs - Rapid Acting	
Insulin Response Enhancers - Biguanide &Dietary Supplement Combination	
Insulin Response Enhancers - Biguanides	
Insulin Response Enhancers - Thiazolidinediones (PPAR-gamma agonists)	
**Antidepressant drugs (generic names)**	**Antipsychotic drugs (generic names)**
amitriptyline	aripiprazole
amoxapine	asenapine
bupropion	brexpiprazole
citalopram	cariprazine
desvenlafaxine	chlorpromazine
doxepin	clozapine
duloxetine	droperidol
escitalopram	fluphenazine
Esketamine	haloperidol
fluoxetine	iloperidone
imipramine	loxapine
ketamine	lumateperone
levomilnacipran	lurasidone
mirtazapine	olanzapine

© 2024 Fung V et al. *JAMA Health Forum.*

nefazodone	paliperidone
nortriptyline	perphenazine
paroxetine	pimozide
protriptyline	quetiapine
sertraline	risperidone
trazodone	thioridazine
trimipramine	thiothixene
venlafaxine	trifluoperazine
vilazodone	ziprasidone
vortioxetine	

© 2024 Fung V et al. *JAMA Health Forum.*

eTable 4. Sample sizes for drug use analyses in 2008 and 2018

	2008				2018			
	No Loss	Temporary Loss	Extended Loss	Reduced Subsidy	No Loss	Temporary Loss	Extended Loss	Reduced Subsidy
Overall Cohort	598,499	33,730	75,680	15,556	670,029	40,314	94,478	13,819
Overall drug Model Cohort	471,918	29,874	67,665	14,254	379,810	35,920	80,435	12,207
Antidiabetes drug model	112,123	6,996	13,979	3,894	106,368	9,907	20,819	3,892
Non-insulin drug model	98,554	5,919	3,406	12,183	92,580	8,378	3,388	18,414
Insulin drug model	36,825	2,746	1,364	4,720	42,039	4,357	1,589	7,349
Antilipid drug model	193,317	10,632	24,090	6,850	187,501	15,620	39,148	6,741
Antidepressant drug model	129,732	9,759	4,773	19,330	132,154	13,764	5,016	27,881
Antipsychotic drug model	38,897	4,076	1,253	5,819	35,252	4,542	1,165	6,309

Note: Beneficiaries with combined loss of LIS and Part D coverage were excluded from drug use analyses due to missing drug use data.

© 2024 Fung V et al. *JAMA Health Forum.*

eTable 5. Percent of beneficiaries with each type of subsidy loss among non-deemed Full LIS

	No. with Full LIS in Dec of prior year	% who Retained Full LIS	% with a Temporary Loss of LIS	% with an Extended Loss of LIS	% with a Reduced Subsidy	% who Disenrolled from Part D after loss of LIS
2008	731,070	81%	5%	10%	2%	2%
2009	699,006	82%	5%	9%	2%	2%
2010	729,008	84%	5%	7%	2%	2%
2011	740,480	79%	5%	10%	3%	3%
2012	767,603	84%	4%	9%	2%	1%
2013	762,749	81%	4%	10%	2%	2%
2014	781,165	81%	5%	9%	2%	3%
2015	783,313	81%	5%	9%	2%	4%
2016	837,158	79%	5%	10%	2%	4%
2017	835,030	78%	5%	11%	2%	5%
2018	862,319	78%	5%	11%	2%	5%

Changes in subsidy status were assessed between Jan-Apr of current year to account for the 3-month grace period.

© 2024 Fung V et al. *JAMA Health Forum.*

eTable 6. Mean months with full, partial, or no subsidy or Part D by subsidy loss categories

	Temporary Loss: n=390,517	Extended Loss: n=821,789	Reduced Subsidy: n=162,111	Disenrolled from Part D: n=269,307
	Mean (SD)	Mean (SD)	Mean (SD)	Mean (SD)
Months with Full LIS	7.5 (3.1)	0.28 (0.86)	0.15 (0.61)	0.23 (0.78)
Months with Partial LIS	0.37 (1.6)	0	11.2 (2.0)	0
Months with No Subsidy	3.3 (3.0)	11.7 (0.87)	0.56 (1.7)	4.2 (2.5)
Months with No Part D	0.79 (1.7)	0	0.12 (0.65)	7.5 (2.6)

© 2024 Fung V et al. *JAMA Health Forum.*

eTable 7. Likelihood of having an annual change in subsidy status by age group and race/ethnicity among non-deemed full LIS recipients (data correspond to Figure 2)

Type of subsidy loss	Beneficiary characteristics	Predicted Probability	95% CI	Odds Ratio	95% CI	p-value
Temporary loss	Age 65-79 (ref)	4.0%	(3.96%, 4%)	1.00		
	Age <65	5.5%	(5.45%, 5.51%)	1.40	(1.39, 1.41)	<.001
	Age 80+	3.9%	(3.87%, 3.94%)	0.93	(0.92, 0.95)	<.001
	Race/ethnicity:					
	White (ref)	4.1%	(4.12%, 4.16%)	1.00		
	AI/AN	5.5%	(5.27%, 5.65%)	1.36	(1.31, 1.41)	<.001
	Asian	4.7%	(4.59%, 4.77%)	1.10	(1.07, 1.12)	<.001
	Black	5.5%	(5.44%, 5.51%)	1.32	(1.31, 1.33)	<.001
	Hispanic	5.3%	(5.27%, 5.36%)	1.25	(1.24, 1.27)	<.001
	Other	4.1%	(3.99%, 4.3%)	0.98	(0.94, 1.02)	0.338
	Unknown	4.3%	(4.07%, 4.48%)	1.07	(1.02, 1.13)	0.011
Extended loss	Age 65-79 (ref)	10.2%	(10.14%, 10.21%)	1.00		
	Age <65	9.4%	(9.36%, 9.43%)	0.94	(0.93, 0.94)	<.001
	Age 80+	7.8%	(7.73%, 7.83%)	0.73	(0.72, 0.73)	<.001
	Race/ethnicity:					
	White (ref)	10.2%	(10.17%, 10.23%)	1.00		
	AI/AN	9.7%	(9.47%, 10%)	0.98	(0.95, 1.01)	0.186
	Asian	8.4%	(8.27%, 8.5%)	0.79	(0.78, 0.81)	<.001
	Black	8.8%	(8.76%, 8.86%)	0.86	(0.85, 0.87)	<.001
	Hispanic	7.8%	(7.77%, 7.88%)	0.75	(0.74, 0.75)	<.001
	Other	9.0%	(8.81%, 9.28%)	0.87	(0.84, 0.89)	<.001
	Unknown	12.5%	(12.16%, 12.85%)	1.27	(1.23, 1.32)	<.001
Reduced subsidy	Age 65-79 (ref)	2.2%	(2.15%, 2.18%)	1.00		
	Age <65	1.8%	(1.78%, 1.81%)	0.84	(0.83, 0.85)	<.001
	Age 80+	1.5%	(1.51%, 1.55%)	0.67	(0.66, 0.68)	<.001
	Race/ethnicity:					
	White (ref)	2.1%	(2.09%, 2.12%)	1.00		
	AI/AN	1.4%	(1.33%, 1.54%)	0.69	(0.65, 0.75)	<.001
	Asian	1.4%	(1.31%, 1.41%)	0.63	(0.6, 0.65)	<.001
	Black	1.6%	(1.6%, 1.64%)	0.76	(0.75, 0.77)	<.001
	Hispanic	1.6%	(1.55%, 1.6%)	0.73	(0.72, 0.74)	<.001
	Other	1.6%	(1.52%, 1.72%)	0.75	(0.70, 0.8)	<.001
	Unknown	1.8%	(1.63%, 1.94%)	0.88	(0.8, 0.96)	0.003
Disenrolled from Part D after losing subsidy	Age 65-79 (ref)	2.8%	(2.8%, 2.84%)	1.00		
	Age <65	3.7%	(3.66%, 3.7%)	1.35	(1.34, 1.36)	<.001
	Age 80+	2.3%	(2.3%, 2.35%)	0.78	(0.77, 0.79)	<.001
	Race/ethnicity:	3.1%	(3.07%, 3.1%)	1.00		

© 2024 Fung V et al. *JAMA Health Forum.*

	White (ref)					
	AI/AN	4.8%	(4.68%, 4.99%)	1.66	(1.60, 1.73)	<.001
	Asian	2.8%	(2.7%, 2.83%)	0.87	(0.84, 0.89)	<.001
	Black	3.4%	(3.34%, 3.4%)	1.10	(1.09, 1.11)	<.001
	Hispanic	3.0%	(2.98%, 3.05%)	0.96	(0.94, 0.97)	<.001
	Other	3.1%	(2.97%, 3.24%)	0.99	(0.94, 1.04)	0.66
	Unknown	3.4%	(3.25%, 3.54%)	1.14	(1.09, 1.2)	<.001
No Loss (ref)	Age 65-79 (ref)	80.9%	(80.81%, 80.9%)			
	Age <65	79.6%	(79.6%, 79.7%)			
	Age 80+	84.5%	(84.39%, 84.53%)			
	Race/ethnicity:					
	White (ref)	80.5%	(80.43%, 80.51%)			
	AI/AN	78.5%	(78.15%, 78.92%)			
	Asian	82.8%	(82.64%, 82.99%)			
	Black	80.7%	(80.66%, 80.8%)			
	Hispanic	82.3%	(82.18%, 82.35%)			
	Other	82.1%	(81.73%, 82.43%)			
	Unknown	78.0%	(77.58%, 78.49%)			

Note: This table displays the predicted probability and odds ratio of having an annual change in subsidy loss vs. retaining subsidy for each group based on a multinomial logistic regression comparing each subsidy group to the group that does not lose the Full LIS. The model is also adjusted for sex, quintile of prior year drug costs, an indicator for moving states, enrollment in MA vs. TM in January, year, and state.

© 2024 Fung V et al. *JAMA Health Forum.*

eTable 8. Secondary analyses: Change in monthly out-of-pocket spending and drug fills after vs. before losing full Part D LIS among non-deemed beneficiaries for insulin and non-insulin diabetes drugs

Out-of-Pocket Costs ($)	Diabetes Drug class	Mean in pre-period ($)	% change (Post vs. Pre)	Coeff ($) (Post vs. Pre)	95% CI	p-value
No Loss: Full LIS (Post) vs. Full LIS (Pre)	Non-Insulin	2.04	-1%	-0.02	(-0.03, -0.02)	<.001
	Insulin	3.19	0%	0.00	(-0.01, 0.01)	0.907
Temporary loss[a]: No LIS (Post) vs. Full LIS (Pre)	Non-Insulin	1.63	468%	7.61	(7.52, 7.70)	<.001
	Insulin	2.75	1026%	28.26	(28, 28.53)	<.001
Extended Loss: No LIS (Post) vs. Full LIS (Pre)	Non-Insulin	1.62	415%	6.74	(6.65, 6.83)	<.001
	Insulin	2.74	1058%	28.98	(28.65, 29.31)	<.001
Reduced Subsidy: Partial LIS (Post) vs. Full LIS (Pre)	Non-Insulin	1.94	187%	3.63	(3.52, 3.73)	<.001
	Insulin	3.06	590%	18.06	(17.72, 18.41)	<.001
	**Drug class**	**Mean in pre-period**	**% change (Post vs. Pre)**	**Coeff (Post vs. Pre)**	**95% CI**	**p-value**
**30-Day Prescription Drug Fills**						
No Loss: Full LIS (Post) vs. Full LIS (Pre)	Non-Insulin	1.1	-1%	-0.014	(-0.016, -0.012)	<.001
	Insulin	0.9	0%	-0.002	(-0.004, 0.000)	0.102
Temporary loss[a]: No LIS (Post) vs. Full LIS (Pre)	Non-Insulin	1.0	-17%	-0.170	(-0.177, -0.163)	<.001
	Insulin	0.8	-33%	-0.274	(-0.282, -0.265)	<.001
Extended Loss: No LIS (Post) vs. Full LIS (Pre)	Non-Insulin	1.1	-17%	-0.178	(-0.183, -0.173)	<.001
	Insulin	0.9	-38%	-0.330	(-0.336, -0.325)	<.001
Reduced Subsidy: Partial LIS (Post) vs. Full LIS (Pre)	Non-Insulin	1.1	-11%	-0.127	(-0.136, -0.117)	<.001
	Insulin	0.9	-24%	-0.225	(-0.237, -0.213)	<.001

Notes: LIS=Low-income subsidy

Monthly outcomes in months covered by Part D; Linear regression models with beneficiary fixed effects adjusted for LIS status, MA vs. TM enrollment, month, and year. These models include those with 12 months of the full LIS in the prior year; models examining drug classes include those with use of these drugs in the prior year (see **eTable 3** for sample sizes).

[a] Includes months with partial LIS.

© 2024 Fung V et al. *JAMA Health Forum.*

eTable 9. Change in drug use, cost, and out-of-pocket spending after vs. before losing Full Part D LIS; additional results for the Reduced Subsidy and Temporary loss groups

30-Day Fills		Mean in pre-period	% change	Coeff	95% CI	p-value
Reduced Subsidy: No LIS (Post) vs. Full LIS (Pre)	All Drugs	5.1	-17%	-0.879	(-0.911, -0.847)	<.001
	DM Drugs	1.3	-22%	-0.299	(-0.321, -0.278)	<.001
	Lipid Drugs	0.9	-17%	-0.149	(-0.163, -0.136)	<.001
	Antidepressants	0.9	-18%	-0.167	(-0.181, -0.153)	<.001
	Antipsychotics	0.9	-27%	-0.234	(-0.255, -0.214)	<.001
Reduced Subsidy: Partial LIS (Post) vs. Full LIS (Pre)	All Drugs	5.1	-9%	-0.446	(-0.46, -0.433)	<.001
	DM Drugs	1.3	-14%	-0.182	(-0.191, -0.172)	<.001
	Lipid Drugs	0.9	-10%	-0.089	(-0.094, -0.083)	<.001
	Antidepressants	0.9	-10%	-0.091	(-0.098, -0.085)	<.001
	Antipsychotics	0.9	-23%	-0.207	(-0.217, -0.196)	<.001
Temporary Loss[a]: No LIS (Post) vs. Full LIS (Pre)	All Drugs	4.0	-15%	-0.580	(-0.59, -0.571)	<.001
	DM Drugs	1.2	-21%	-0.255	(-0.263, -0.248)	<.001
	Lipid Drugs	0.8	-16%	-0.126	(-0.131, -0.122)	<.001
	Antidepressants	0.9	-14%	-0.120	(-0.124, -0.115)	<.001
	Antipsychotics	0.8	-19%	-0.150	(-0.156, -0.143)	<.001
Temporary Loss: Full LIS (Post) vs. Full LIS (Pre)	All Drugs	4.0	1%	0.037	(0.028, 0.046)	<.001
	DM Drugs	1.2	-4%	-0.045	(-0.052, -0.038)	<.001
	Lipid Drugs	0.8	-7%	-0.055	(-0.059, -0.051)	<.001
	Antidepressants	0.9	-6%	-0.048	(-0.052, -0.044)	<.001
	Antipsychotics	0.8	-7%	-0.055	(-0.061, -0.049)	<.001
Out-of-Pocket Costs		Mean in pre-period	% change	Coeff	95% CI	p-value
Reduced Subsidy: No LIS (Post) vs. Full LIS (Pre)	All Drugs	9.9	669%	66.4	(66, 66.7)	<.001
	DM Drugs	2.9	707%	20.2	(19.8, 20.6)	<.001
	Lipid Drugs	1.7	447%	7.6	(7.5, 7.8)	<.001
	Antidepressants	1.57	539%	8.45	(8.33, 8.56)	<.001
	Antipsychotics	2.18	1598%	34.89	(34.08, 35.69)	<.001
Reduced Subsidy: Partial LIS (Post) vs. Full LIS (Pre)	All Drugs	9.9	196%	19.5	(19.3, 19.7)	<.001
	DM Drugs	2.9	351%	10.0	(9.9, 10.2)	<.001
	Lipid Drugs	1.7	156%	2.7	(2.6, 2.7)	<.001
	Antidepressants	1.57	148%	2.32	(2.27, 2.38)	<.001
	Antipsychotics	2.18	487%	10.64	(10.24, 11.04)	<.001
Temporary Loss[a]: No LIS (Post) vs. Full LIS (Pre)	All Drugs	7.5	700%	52.7	(52.5, 52.9)	<.001
	DM Drugs	2.6	746%	19.1	(19, 19.3)	<.001
	Lipid Drugs	1.4	411%	5.6	(5.5, 5.6)	<.001

© 2024 Fung V et al. *JAMA Health Forum.*

	Antidepressants	1.38	484%	6.69	(6.63, 6.74)	<.0001
	Antipsychotics	2.00	1770%	35.47	(35.03, 35.91)	<.001
Temporary Loss: Full LIS (Post) vs. Full LIS (Pre)	All Drugs	7.5	-16%	-1.2	(-1.4, -1)	<.001
	DM Drugs	2.6	-22%	-0.6	(-0.7, -0.4)	<.001
	Lipid Drugs	1.4	-4%	0.0	(-0.1, 0)	0.014
	Antidepressants	1.38	-7%	-0.09	(-0.14, -0.04)	<.001
	Antipsychotics	2.00	-104%	-2.09	(-2.5, -1.67)	<.001

[a] Includes months with partial LIS

© 2024 Fung V et al. *JAMA Health Forum.*
