# Supplementary material for: Associations Between Annual Medicare Part D Low-Income Subsidy Loss and Prescription Drug Spending and Use
Source: JAMA Health Forum. 2024 Feb 2;5(2):e235152. doi: 10.1001/jamahealthforum.2023.5152 (PMC10837747; doi:10.1001/jamahealthforum.2023.5152)
Supplement: Supplement 2. — Data Sharing Statement [file jamahealthforum-e235152-s002.pdf]

Data Sharing Statement

Fung. Associations Between Annual Medicare Part D Low-Income Subsidy Loss and Prescription Drug Spending and Use. *JAMA Health Forum*. Published February 02, 2024. doi:10.1001/jamahealthforum.2023.5152

Data
Data available: Yes
Data types: Deidentified participant data
How to access data: Data can be accessed upon application to the Centers for Medicare and Medicaid Services.
When available: With publication

Supporting Documents
Document types: None

Additional Information
Who can access the data: Researchers whose proposed use of the data has been approved
Types of analyses: For specified purposes
Mechanisms of data availability: Approval of Proposal and with Signed Data Access Agreement
